# Immunomodulatory Effects of Perioperative Dexmedetomidine in Ovarian Cancer: An *In Vitro* and Xenograft Mouse Model Study

**DOI:** 10.3389/fonc.2021.722743

**Published:** 2021-10-07

**Authors:** Seokyung Shin, Ki Jun Kim, Hye Jeong Hwang, Sewon Noh, Ju Eun Oh, Young-Chul Yoo

**Affiliations:** ^1^ Department of Anesthesiology and Pain Medicine, Severance Hospital, Anesthesia and Pain Research Institute, Yonsei University College of Medicine, Seoul, South Korea; ^2^ Anesthesia and Pain Research Institute, Yonsei University College of Medicine, Seoul, South Korea

**Keywords:** dexmedetomidine, immunomodulation, ovarian cancer, surgical stress response, sympathetic nervous system

## Abstract

**Background:**

The surgical stress response (SSR) causes immunosuppression which may cause residual tumor growth and micrometastasis after cancer surgery. We investigated whether dexmedetomidine affects cancer cell behavior and immune function in an ovarian cancer xenograft mouse model.

**Methods:**

The effect of dexmedetomidine on cell viability and cell cycle was assessed using SK-OV-3 cells at drug concentrations of 0.5, 0.1, 5, and 10 µg mL^-1^. BALB/c nude mice were used for the ovarian cancer model with the Dexmedetomidine group (n=6) undergoing surgery with dexmedetomidine infusion and the Control group (n=6) with saline infusion for 4 weeks. Natural killer (NK) cell activity, serum proinflammatory cytokines, and cortisol were measured at predetermined time points and tumor burden was assessed 4 weeks after surgery.

**Results:**

Dexmedetomidine had no effect on cell viability or cell cycle. Following a sharp decrease on postoperative day (POD) 1, NK cell activity recovered faster in the Dexmedetomidine group with significant difference vs. the Control group on POD 3 (*P*=0.028). In the Dexmedetomidine group, cortisol levels were lower on POD 3 (*P*=0.004) and TNF-α levels were lower at 4 weeks after surgery (*P*<0.001) compared to the Control group. The Dexmedetomidine group showed lower tumor burden at 4 weeks vs. the Control group as observed by both tumor weight (*P*<0.001) and the *in vivo* imaging system (*P*=0.03).

**Conclusions:**

Dexmedetomidine infusion may improve ovarian cancer surgery outcome by suppressing the SSR and stress mediator release. Further studies are needed to elucidate the mechanisms by which dexmedetomidine acts on cancer and immune cells.

## Introduction

Although surgical excision remains the mainstay of treatment for solid tumors, perioperative immunosuppression may adversely promote residual tumor growth and micrometastasis after surgery (1, 2). Immunosuppression is a feature of the perioperative stress response which is associated with the hyperactivation of the sympathetic nervous system (SNS) and release of acute-phase proteins (3, 4). The deleterious effect of physiologic stress and SNS activation on cancer biology has been widely investigated (5). A key player in this process is the natural killer (NK) cell, which is critical for anti-tumor immunity, but becomes suppressed in proinflammatory and adrenergic stressor states (6).

As a potential method to alleviate the surgical stress response (SSR) and reduce immunosuppression, we focused on dexmedetomidine, a highly selective α2 adrenergic agonist well known for its analgesic properties and also the ability to suppress SNS activity (7). Moreover, NK cells express α2 adrenoreceptors (8, 9), and clonidine has been found to enhance NK cell cytotoxicity (9). Dexmedetomidine was reported to attenuate perioperative stress and inflammation induced by surgical trauma and to protect immune function of surgical patients in a recent systematic review (10). Interestingly, an *in vivo* study found dexmedetomidine to promote metastasis in breast, lung, and colon cancer in rodent models and found it to be dose-dependently deleterious (11). These conflicting results may be due to different cancer types expressing different adrenoreceptors or the use of varying doses of dexmedetomidine.

To further investigate the effect of dexmedetomidine on cancer cell behavior and immune function, we aimed to study 1) the effect of dexmedetomidine on ovarian cancer cell viability and its cell cycle *in vitro* 2) the effect of dexmedetomidine on NK cell cytotoxicity, cortisol and inflammatory cytokines levels in an ovarian cancer micrometastasis mouse model. Further, we investigated 3) whether the effect of dexmedetomidine would lead to a significant difference in tumor burden and metastasis *in vivo*.

## Materials and Methods

### Cell Culture and Lentiviral Particles Transduction

The SK-OV-3 human ovarian cancer cell line (12) was purchased from the American Type Culture Collection (Manassas, VA, USA). Cells were cultured in Roswell Park Memorial Institute (RPMI)-1640 medium (Hyclone, Logan, Utah, USA) supplemented with 10% heat-inactivated fetal bovine serum (FBS, Gibco), L-glutamine, 100 IU mL^-1^ penicillin, and 100 μg mL^-1^ streptomycin at 37°C in a humidified atmosphere of 5% CO_2_. SK-OV-3 cells were seeded onto 24-well plates at a density of 2×10^4^ cells per well in complete RPMI medium and incubated for 15-20 h. Redifect Red-Flu-Puromycin lentiviral particles (PerkinElimer, MA, USA) were thawed on ice. The culture medium for SK-OV-3 cells were replaced with 0.5 mL of fresh complete medium containing hexadimethrine bromide at a final concentration of 5 μg mL^-1^. Thawed viral particles were added to the cells directly at a multiplicity of infection of 50. After 24 h, 500 μL of fresh pre-warmed complete culture medium was added and cells were incubated for 24 h. Transduced cells were selected with 2 μg mL^-1^ puromycin in fresh complete culture medium. Transduction efficiency was also determined by using the IVIS *In Vivo* Imaging System (Perkin Elmer, USA). SK-OV-3-Luc cells were assayed for luciferase expression.

### Cell Viability and Cell Cycle Analysis

SK-OV-3 cells were plated onto 96-well plates at a density of 5×10^3^ cells per well and divided into control and treatment groups. After 24h, dexmedetomidine (Hospira Inc., Rocky Mount, NC, USA) was added to the treatment group in four concentrations of 0.5, 0.1, 5 and 10 μg mL^-1^, whereas phosphate buffered saline (PBS) was added to the Control group. Cell viability was assessed after 48 h using the EZ-Cytox Cell Viability Assay Kit (Dogen, Seoul, South Korea). Absorbance at 450 nm was measured in the experimental groups using a plate reader. Experiments were performed in six biological replicates and repeated three times.

For cell cycle analysis, SK-OV-3 cells were detached with 0.25% trypsin-EDTA (Gibco) and fixed in 70% ethanol for 30 min at 4°C. Cells were washed twice with cold PBS and treated with 0.25 mL RNase (10 mg mL^-1^) in 50 mL of PBS for 20 min at 37°C; 5 mL of propidium iodide solution (ThermoFisher Scientific, Waltham, MA, USA) in 50 mL of PBS was then added and mixed well. Thereafter, cells were incubated for 30 min in the dark at room temperature. The labelled cells were analyzed using a BD FACScan flow cytometer (Becton Dickinson Biosciences, Franklin Lakes, NJ, USA).

### Xenograft Models and Experimental Design

All animal procedures were approved by the Animal Care and Use Committee of Yonsei University Health System (IACUC 2017-0254). Seven-week-old female BALB/c nude mice weighing 18-20 g were experimented in accordance with the Guide for the Care and Use of Laboratory Animals (US National Institutes of Health).

For mouse ovarian cancer xenograft models, BALB/c nude mice were subcutaneously injected with SK-OV-3-Luc cells (5×10^6^ cells in 0.1 mL PBS) in the dorsal skin. Tumors were allowed to grow into visible masses for 10 days and then excised in sizes of 2×3×2 mm^3^. Eighteen nude mice were randomly assigned to either the Sham group (*n*=6), the Control group (*n*=6), and the Dexmedetomidine group (*n*=6). The Sham group received only a skin incision. The mice of the remaining two groups underwent left ovariectomy and pre-grown tumor masses were sutured in place followed by dissemination of SK-OV-3-Luc cells in concentrations of 5×10^6^ in 0.1 mL of PBS. Surgical procedures were performed under isoflurane anesthesia.

To evaluate the therapeutic response of the metastatic ovarian tumor xenografts to dexmedetomidine, a micro-osmotic pump system (ALZET model 1004, USA) was implanted subcutaneously in the left flank of the mice in the Control group and the Dexmedetomidine group. The drug-loaded pump systems were assembled for infusion according to the manufacturer’s instructions and implanted simultaneously with ovariectomy. The Dexmedetomidine group received an infusion of dexmedetomidine 12 μg kg^-1^ day^-1^ at a flow rate of 0.11 μL h^-1^ while the Control group received the same volume of normal saline. The infusion rate of dexmedetomidine was based on clinically recommended infusion rates in humans. Treatments were delivered continuously for 4 weeks with no signs of apparent sedation or procedural mortality. After 4 weeks, imaging was performed with an IVIS imaging system, and the mice were sacrificed. Tumor burden was assessed by weighing excised tumor masses.

### Natural Killer Cell Activity

Whole blood samples were collected from the SK-OV-3-Luc xenograft mice right before surgery (day 0) and postoperative day (POD) 1, 3 and 5. NK cell activity was determined using the NK Vue Gold kit (ATGen, Seongnam-si, Korea) which measures the level of interferon-gamma (IFN-γ) released from activated NK cells using sandwich enzyme immunoassay.

### Enzyme-Linked Immunosorbent Assay (ELISA)

Levels of serum tumor necrosis factor alpha (TNF-α), interleukin-6 (IL-6), and interleukin-1 beta (IL-1β) were analyzed at 4 weeks after surgery and cortisol levels were measured on POD 3 by using ELISA commercial kits from R&D Systems (Minneapolis, MN, USA) according to the manufacturer’s instructions.

### *In Vivo* Bioluminescence Imaging

Images were acquired using the IVIS Spectrum and analyzed using Living Image 4.5.5 software. To generate bioluminescence signals, D-luciferin (potassium salt, PerkinElmer Inc.) 150 mg kg^-1^ was intraperitoneally injected into mice prior to imaging. Animals were then anaesthetized with 2% isoflurane and placed in the imaging chamber. All fluorescence images were acquired with a 7 min exposure. For quantitative comparison, regions of interests (ROIs) were drawn over the tumor and the results were expressed as the mean ± standard deviation for a group of seven animals.

### Statistical Analysis

All data from the *in vitro* and *in vivo* experiments are expressed as mean (standard deviation) and were analyzed using student’s t-test, one-way analysis of variance (ANOVA) followed by Bonferroni’s multiple comparison test or repeated measures ANOVA. *P*<0.05 was considered statistically significant.

## Results

### Cell Viability and Cell Cycle *In Vitro*

**Figure 1:**
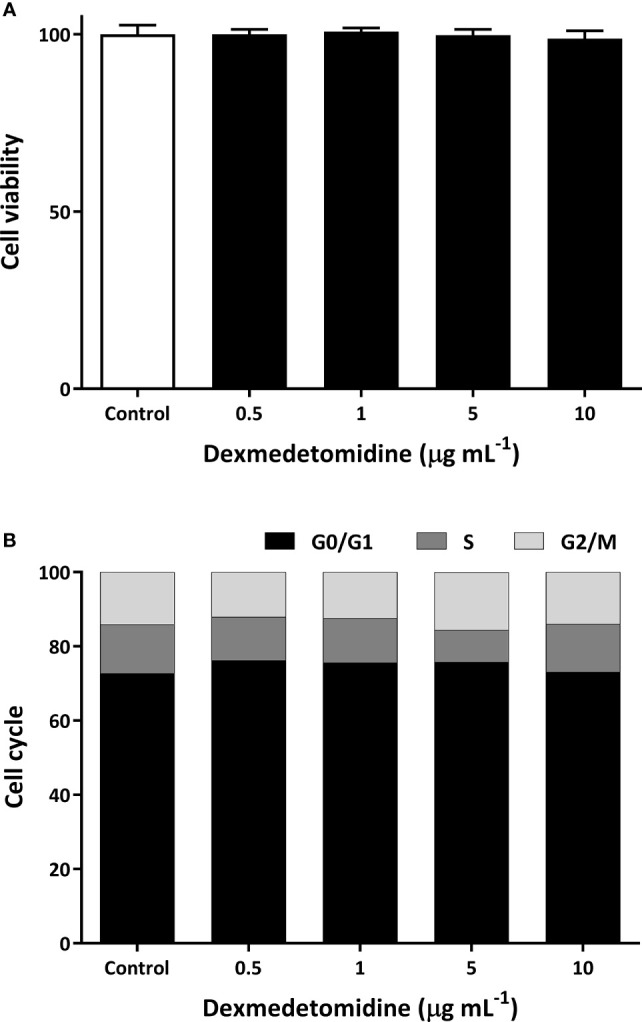
Cell viability **(A)** and cell cycle **(B)** of SK-OV-3 human ovarian cancer cells with dexmedetomidine at 0.5, 1, 5, and 10 μg mL^-1^. Error bars represent standard deviation.

As shown in **Figure 1A**, we were not able to see any effects on cell viability with dexmedetomidine. Compared to control, there were no changes in cell morphology and viability at four different concentrations of dexmedetomidine ranging from 0.5 µg mL^-1^ to 10 µg mL^-1^. Similarly, there were no effects on cell cycle compared to control (**Figure 1B**) at the four different concentrations of dexmedetomidine. These results show that dexmedetomidine has no effect on ovarian cancer cell viability or cell cycle *in vitro*.

### Natural Killer Cell Activity and Cortisol ELISA *In Vivo*

**Figure 2:**
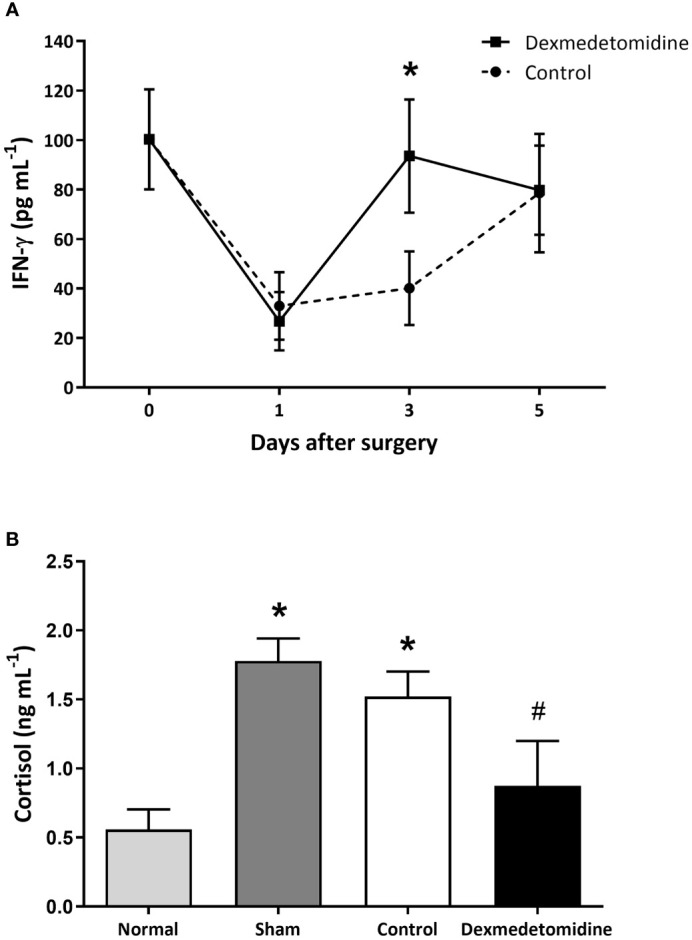
**(A)** NK cell activity measured by IFN-γ released from activated NK cells in mice 1, 3, and 5 days after surgery. *P < 0.05 compared to Control. **(B)** Serum cortisol levels in mice 3 days after surgery. *P < 0.05 compared to Normal. ^#^ *P* < 0.05 compared to Sham and Control group. Error bars represent standard deviation.

NK cell activity was measured on days 0, 1, 3, and 5 after surgery. NK cell activity decreased on POD 1 in both the control and dexmedetomidine treated mice. NK cell activity start increasing in both groups on POD 3, but a greater increase was seen in mice that received dexmedetomidine with a significant difference between the two groups (*P*=0.028). NK cell activity recovered to similar levels in both groups on POD 5 (**Figure 2A**). Serum cortisol levels measured on POD 3 showed cortisol levels to be significantly lower in the Dexmedetomidine group compared to the Control group (*P*=0.004). There was no difference between the Dexmedetomidine group and normal mice (**Figure 2B**).

### Blood Cytokine Levels Measured With ELISA

**Figure 3:**
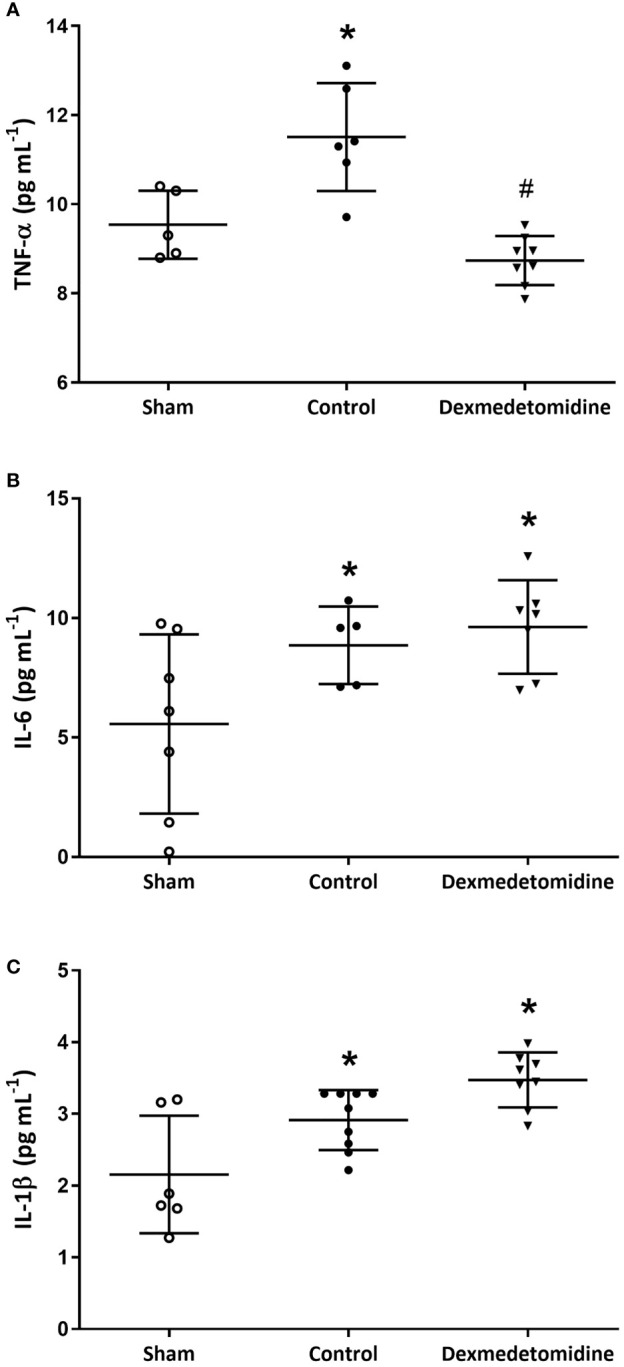
**(A)** TNF-α levels at 4 weeks after surgery. *P < 0.05 compared to Sham. ^#^ *P* < 0.05 compared to Sham and Control group. **(B)** IL-6 levels at 4 weeks after surgery. *P < 0.05 compared to Sham. **(C)** IL-1β levels at 4 weeks after surgery. *P < 0.05 compared to Sham. Error bars represent standard deviation.

Blood cytokine levels measured after sacrificing the mice 4 weeks after surgery showed TNF-α to be significantly lower in the Dexmedetomidine group compared to the Control group (*P*<0.001). However, IL-6 and IL-1β were similar between the Control and Dexmedetomidine groups, with both being significantly greater than the Sham group (**Figure 3**).

### Tumor Growth and Burden

**Figure 4:**
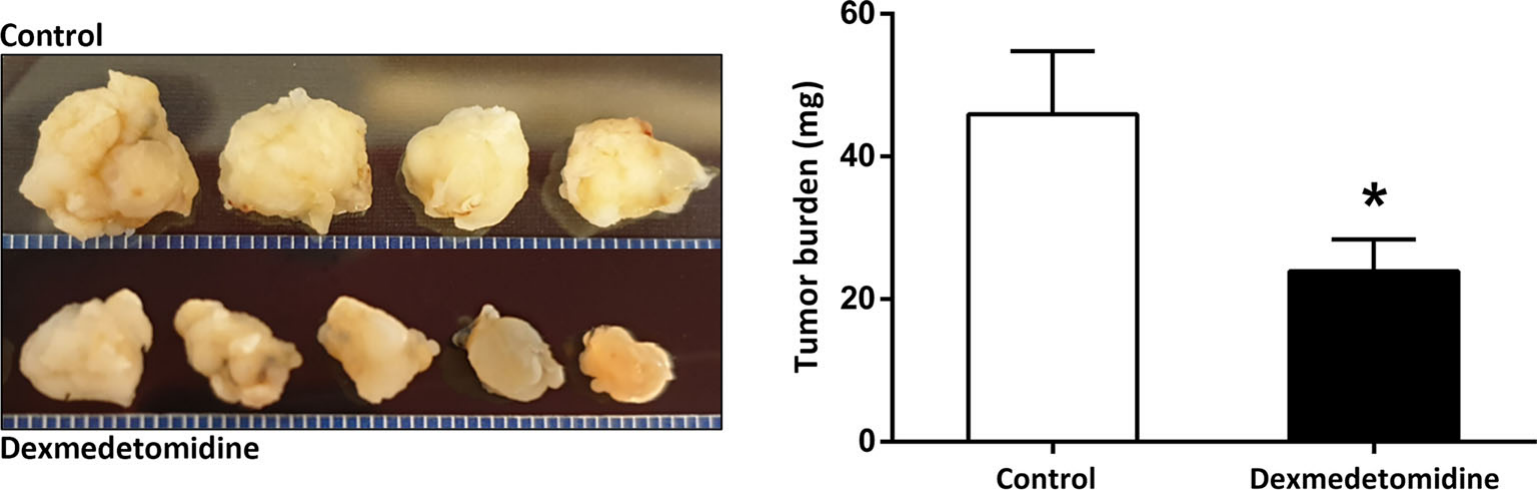
Tumor size and weight at 4 weeks after surgery in the Control and Dexmedetomidine groups. *P < 0.05 compared to Control. Error bars represent standard deviation.

Tumor growth observed over 4 weeks showed slower growth and smaller tumor size with dexmedetomidine compared with control. Tumor weight was significantly smaller in mice treated with dexmedetomidine compared to control (*P*<0.001) (**Figure 4**).

### Tumor Growth With *In Vivo* Imaging System

**Figure 5:**
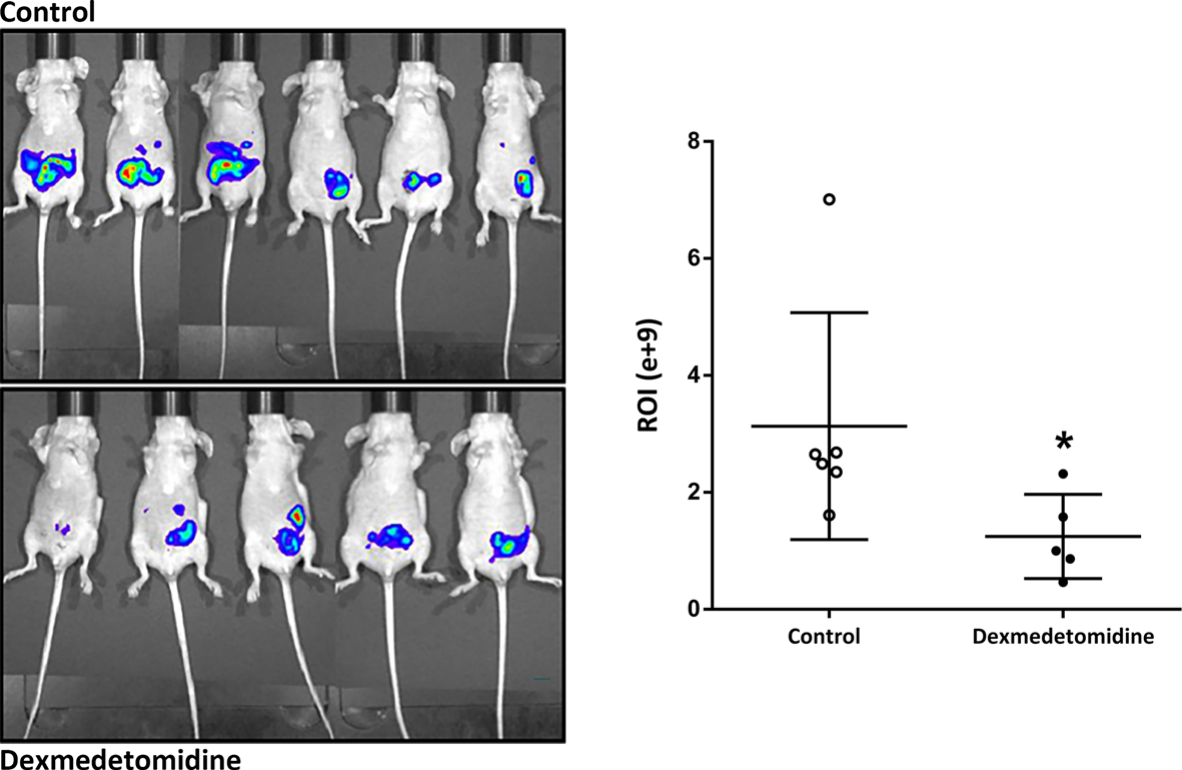
Cancer cell expression observed with *in vivo* Imaging system at 4 weeks after surgery. *P < 0.05 compared to Control. Error bars represent standard deviation.

In the *in vivo* micrometastasis mice model, decreased cancer cell expression was observed in mice treated with dexmedetomidine at 4 weeks after surgery with IVIS. Quantified results were able to show a significantly lower expression of cancer cells in the Dexmedetomidine group compared to the Control group (*P*=0.03) (**Figure 5**).

## Discussion

It is ironic and worrisome that surgical excision of solid tumors may unintentionally accelerate cancer recurrence. The SSR leads to a phase of postoperative immunosuppression during the first three weeks, which has been described as the “immunological window of opportunity” (13). As a potential method to alleviate such immunosuppression after surgery, we studied the effects of dexmedetomidine in an ovarian cancer micrometastasis mouse model, and found that while dexmedetomidine had no effect on cancer cell viability or cell cycle *in vitro*, it led to lower cortisol levels and faster recovery of NK cell activity *in vivo* postoperatively. Further, mice given dexmedetomidine showed significantly lower tumor burden and TNF-α levels compared to the Control group 4 weeks after surgery, suggesting a possible role for dexmedetomidine to abrogate the SSR and therefore alleviate immunosuppression during the perioperative window.

### Dexmedetomidine and the Stress Response

Perioperative stress induces the secretion of stress-related mediators such as catecholamine and cortisol from SNS or hypothalamic-pituitary-adrenocortical (HPA) axis activation. Catecholaminergic signals are thought to be involved not only with tumor growth and progression, but also with metastasis and resistance to programmed cell death of tumor cells (14, 15). The role of neural regulation in tumor growth and metastasis has been established in different types of malignancies including ovarian cancer (16). Cortisol is known to act synergistically with adrenergic cellular mechanisms, potentiating adrenergically induced increases in cyclic AMP in tumor cells, therefore enhancing tumor growth (17, 18).

The adrenal medulla plays a central role in the secretion of systemic catecholamines where chromaffin cells release catecholamines into the bloodstream in response to acute and chronic stress. While this is mainly controlled by neural and humoral mechanisms, the secretory products themselves may also act in an autocrine or paracrine manner. A typical example are the α2 adrenoreceptors in chromaffin cells, which respond to catecholamines with negative feedback to self-limit further secretion (19). As a highly selective α2 agonist, dexmedetomidine exhibits a specificity of 1620:1 (α2:α1) which is almost 8 times greater than clonidine (20). Patients that received intraoperative dexmedetomidine showed lower levels of epinephrine, norepinephrine and cortisol during the perioperative period (10, 21, 22). In fact, intraoperative dexmedetomidine infusion was reported to be as effective as epidural anesthesia in reducing the SSR (23). Although we did not evaluate catecholamine levels in our study, the cortisol levels of mice treated with dexmedetomidine were not only significantly lower compared to the Control group, but also comparable to the cortisol levels in normal mice.

NK cells play a critical role in antitumor surveillance, which is unfortunately severely impaired during the perioperative period due to the release of stress mediators (24). While both groups experienced a dramatic decline in NK cell activity on POD 1 in our study, the Dexmedetomidine group showed NK cell activity to be restored to nearly baseline levels on POD 3 while the Control group showed almost no recovery. Based on these findings, a possible explanation for our results may be that dexmedetomidine was able to suppress the SSR and release of stress hormones, leading to a prompter restoration of NK cell activity to a degree that the growth of the ovarian cancer cells was ultimately reduced. However, it should be kept in mind that what we’ve observed in our study is the net result of a highly complex regulatory system that involves crosstalk between the autonomic nervous system and HPA axis in response to a stressor. Although the focus of this study is the mechanism by which dexmedetomidine may have affected the SNS and therefore cancer outcome in our animal model, we are not able to analyze the overall interaction and sequential feedback between the SNS, parasympathetic nervous system, and the HPA axis through our results.

### Adrenoreceptors Expressed in Ovarian Cancer Cells

Another mechanism to consider is the possibility of dexmedetomidine acting directly on α2 adrenoreceptors expressed in cancer cells. In breast cancer, both human (25) and mouse mammary cell lines (26) have been found to express functional α2 adrenoceptors. In humans, α2 adrenoreceptors were found to be associated with an increase in cell proliferation (25). Similarly, a significant enhancement of mouse mammary tumor growth was observed with clonidine (26).

The main receptor identified in ovarian cancer cells is the β2 adrenergic receptor through which catecholaminergic signals are predominantly mediated (27). Surgical stress was shown to promote tumor growth through increased angiogenesis and vascular endothelial growth factor (VEGF) production *via* β adrenoreceptors on ovarian cancer cells. Moreover, remote surgical stress led to increased tumor growth in the ovary, and such effects were blocked by a nonspecific β blocker (28). As of now, however, whether α adrenoreceptors are expressed in ovarian cancer cells is not clear. If in fact ovarian cancer cells do express α2 adrenoreceptors, dexmedetomidine may have directly affected SK-OV-3 cells *in vitro*. Based on the fact that we were not able to observe any changes in ovarian cancer cell viability or cell cycle with dexmedetomidine, it seems unlikely that dexmedetomidine affected cell growth directly *via* α2 adrenoreceptors. Rather, the aforementioned ability of dexmedetomidine to alleviate the SSR may have acted indirectly on β adrenoreceptors of the ovarian cancer cells, resulting in reduced tumor burden *in vivo*.

### α2 Adrenoreceptors Expressed in NK Cells

There is also the possibility of dexmedetomidine acting directly on α2 adrenoreceptors expressed on immune cells. As mentioned above, it has been shown in animal models and clinical studies that NK cell cytotoxicity is suppressed immediately after surgery (29, 30).

NK cells have been reported to express both α and β adrenoreceptors (8, 9) and in rats, activation of either α1- and α2- adrenoreceptors were found to augment NK cytotoxicity (9). This direct action of dexmedetomidine may have acted additively to its systemic suppression of the SSR, resulting in the faster recovery of NK cell activity in the Dexmedetomidine group.

Previously, dexmedetomidine was found to significantly suppress TNF- α and IL-6 levels in patients undergoing laparoscopic ovarian resection for cancer (31). Our results are in line with this previous study in that NK cell activity recovered faster after laparotomy while TNF-α levels were lower with significantly lower tumor burden in mice treated with dexmedetomidine.

### The Current Evidence

The existing evidence on dexmedetomidine and cancer are conflicting, including opposing results between animal studies and human studies. One of the earlier animal studies reported that dexmedetomidine inhibited antitumor immunity due to a decreased Th1/Th2 ratio in thymoma cells, and stated that this result “was a surprise” (32). Of note, dexmedetomidine was not administered as a continuous infusion but as twice-daily bolus doses in mice for a week in this study. Similarly, dexmedetomidine was found to have deleterious effects on tumor metastasis in rodent models of breast, lung, and colon cancer (11). Here, the administration of yohimbine was found to prevent the metastasis promoting effects of dexmedetomidine, which also shows that these effects are mediated through α2 adrenoreceptors. However, the dose of dexmedetomidine used in this study far exceeded the clinically recommended infusion dose and was soon met with an opposing report where clonidine was not found to be associated with worse outcome in breast and lung cancer patients (33).

We are still at an early stage of investigating the effect of α-adrenoreceptors on cancers of various organs. At this point, even the effect of the relatively well-studied β adrenergic antagonists seem to be variable and tumor specific (34). α2 agonists such as dexmedetomidine require future studies that explore their effects in different types of malignancies at clinical doses and infusion periods.

### Limitations

This study has several limitations. While the lower cortisol levels in the Dexmedetomidine group reflect lesser SNS activation, a serial measurement over the course of 5 days would have allowed more insight into the action of dexmedetomidine in cancer surgery. In the same vein, a serial measurement of blood cytokine levels would have offered relevant data to our study. One of the main mechanisms through which anesthetics may directly affect outcome after cancer surgery is the regulation of HIF levels (35). Increases in HIF-1α and HIF-2α have both been suggested to be associated with ovarian carcinoma progression (36). In the same respect, VEGF plays an important role in the pathogenesis of ovarian cancer by contributing to the development of peritoneal carcinomatosis, and its inhibition has been shown to suppress tumor growth and invasion (37). It would be interesting to see whether the use of dexmedetomidine in our animal model will affect HIF-1 expression and intraperitoneal concentrations of VEFG and whether this correlates with differences in tumor burden between groups. Most importantly, our study has the inherent limitation of being an animal study that cannot be directly translated into replications in a human trial. As mentioned above, we employed an infusion rate that is within the clinically recommended dose, which is significantly lower that the doses used in previous studies that reported problematic effects of dexmedetomidine on tumor growth and metastasis (11). Considering the fact that we maintained dexmedetomidine infusion for 4 weeks, our results suggest that dexmedetomidine is probably at least safe as a perioperative adjuvant drug in ovarian cancer when used within clinical recommendations, and has the potential to be an effective immunomodulatory drug during the perioperative period.

### Conclusion

In conclusion, we found dexmedetomidine infusion to have a potentially positive effect on surgical outcome in a mouse model of ovarian cancer. This may be due to several reasons, as dexmedetomidine can be expected to act on different levels. Systemically on a neuroendocrine and paracrine level, dexmedetomidine is able to alleviate the SSR and therefore inhibit the release of stress mediators. This in turn may exert indirect effects on ovarian cancer cells and NK cells. There also lies the possibility of direct action on adrenoreceptors expressed in NK cells. Further studies are needed to elucidate potential mediating mechanisms and whether these effects are tumor-specific.

## Data Availability Statement

The raw data supporting the conclusions of this article will be made available by the authors, without undue reservation.

## Ethics Statement

The animal study was reviewed and approved by The Animal Care and Use Committee of Yonsei University Health System.

## Author Contributions

SS: study design, data collection, data analysis, and writing the manuscript. KJK: study design and data analysis. HJH: data collection and data analysis. SN: data collection and data analysis. JEO: study design, data collection, and data analysis. Y-CY: study design, data analysis, and writing the manuscript. All authors contributed to the article and approved the submitted version.

## Funding

This study was supported by NRF-2017R1C1B5015221 Ministry of Science and ICT, South Korea.

## Conflict of Interest

The authors declare that the research was conducted in the absence of any commercial or financial relationships that could be construed as a potential conflict of interest.

## Publisher’s Note

All claims expressed in this article are solely those of the authors and do not necessarily represent those of their affiliated organizations, or those of the publisher, the editors and the reviewers. Any product that may be evaluated in this article, or claim that may be made by its manufacturer, is not guaranteed or endorsed by the publisher.
